# CFTI5Med, the new release of the catalogue of strong earthquakes in Italy and in the Mediterranean area

**DOI:** 10.1038/s41597-019-0091-9

**Published:** 2019-06-03

**Authors:** Emanuela Guidoboni, Graziano Ferrari, Gabriele Tarabusi, Giulia Sgattoni, Alberto Comastri, Dante Mariotti, Cecilia Ciuccarelli, Maria Giovanna Bianchi, Gianluca Valensise

**Affiliations:** 1 Istituto Nazionale di Geofisica e Vulcanologia, Rome, Italy; 2 EEDIS, Eventi Estremi e Disastri, Centro euro-mediterraneo di documentazione, Bologna, Italy

**Keywords:** Seismology, Natural hazards

## Abstract

A key element for assessing seismic hazard and risk is the availability of a comprehensive dataset on past earthquakes. Here we present the rationale, structure and contents of CFTI5Med (10.6092/ingv.it-cfti5), the 2018 version of the Catalogue of Strong Earthquakes in Italy: a large multidisciplinary effort including historians, seismologists and geologists. It was conceived in 1989, following the inception of GIS technology, and first published in 1995 to offer a full account of Italy’s strongest earthquakes, of their territorial impact and associated social and economic upheaval. Subsequent versions (1997, 2000, 2007) entailed a fine tuning of research methodologies, included additional research on Italian earthquakes, and were extended to large earthquakes of the Mediterranean area. CFTI5Med comprised an opportunity to streamline the structure of the Catalogue database and propose a renovated user interface. The new front-end (1) grants an easier, intuitive access to the data, including earthquake effects on the environment, and (2) allows all data to be displayed jointly with relevant topographic, geological and seismological overlays published as web services.

## Abstract (summary)

**Table:** 

Design Type(s)	data integration objective • source-based data analysis objective
Measurement Type(s)	earthquake
Technology Type(s)	digital curation
Factor Type(s)	geographic location • temporal_interval
Sample Characteristic(s)	Mediterranean Region

Machine-accessible metadata file describing the reported data (ISA-Tab format)

## Background & Summary

Understanding any natural, potentially adverse phenomena requires knowledge of their history and territorial impact. Whether it is geological or human history (or a combination thereof) depends on the characteristic return period of the phenomena under scrutiny. Learning about major global climate changes and sea level fluctuations can hardly rely on historical evidence, since these processes occur at time scales in the order of 100,000 years; but geological evidence, rock deposits and landforms, will help filling the gap. Conversely, we can rely on written sources - and occasionally even archaeological evidence - to investigate earthquakes, volcanic eruptions, tsunami, floods and major landslides, all of which occur at timescales ranging from a few decades to a few millennia. Eruptions, tsunami and landslides may leave a discernible geological record even in such a short timescale - as in the case of Pompeii and the Vesuvius, or the 1963 Vajont landslide in the Italian Alps - but earthquakes generally do not, as their characteristic geological signature is generally rather subdued.

The tradition of keeping track of such phenomena dates back to the Greek and Roman civilizations. Ancient earthquakes went largely unnoticed when they occurred in rural or uninhabited areas, but not when they hit the first Mediterranean megalopolis, thus turning into full-fledged *catastrophes*.

Not surprisingly, earthquakes have left a strong imprint on the Italy’s built environment, heritage and traditions. Italian scholars learned soon that mitigating seismic risk requires understanding where and why earthquakes occur. Major advancements were spurred by catastrophic events that attracted the interest of erudite contemporaries, such as the central-southern Italy earthquakes of 1456. Today this vast, inherently multidisciplinary field lying at the foundations of most investigations into seismic hazard and risk is referred to as Historical Seismology.

In 1873 and 1883, respectively, Italian earth scientists Michele Stefano de Rossi and Giuseppe Mercalli proposed empirical scales for classifying the *intensity* of earthquake effects objectively, turning Historical Seismology into quantitative science. Intensity allowed different events to be compared in terms of location and overall severity.

In 1901 Mario Baratta^1^ published a catalogue including nearly 1,400 Italian earthquakes between the 1^st^ century AD and 1898. Alfonso Cavasino^2^ (1935) updated and systematised Baratta’s work, eventually leading to the first fully computerised catalogue in 1973^3^.

Spurred by the need to re-assess the seismic hazard of potential nuclear sites, in the 1980s Italian Historical Seismology entered its Golden Age. Between 1983 and 1988 specific investigations were appointed to SGA Storia Geofisica Ambiente private firm by ENEL (Italy’s electricity company). In 1985 Daniele Postpischl^4^ published a “consensus” catalogue based on the results obtained by research groups of Italy’s Progetto Finalizzato Geodinamica (PFG). In-depth studies on several large Italian earthquakes appeared in the *Atlas of isoseimal maps*^5^, a crucial tool in highlighting the importance of using original historical sources to investigate past earthquakes. Earthquakes were no longer represented only by an epicentre, but were regarded as complex phenomena to be investigated through their global territorial impact.

When Italy dropped its plans for producing nuclear energy following the Chernobyl accident (1986), Historical Seismology studies resumed as part of the mandate of Istituto Nazionale di Geofisica (ING). In 1995 ING released the first version of the Catalogue of Strong Earthquakes in Italy (originally *Catalogo dei Forti Terremoti in Italia*, or CFTI: hereinafter “Catalogue”), a “new-generation” compilation summarising all research results available to date^6^. Its second release^7^ (1997) was paralleled by the Internet-based compilation of Italy’s *National group for defence against earthquakes*^8^ (GNDT). Both compilations were used for assembling the *Italian Parametric Earthquake Catalogue*^9^.

The record of Italian historical seismicity is probably the most extensive worldwide, making it of potential interest for a wider international readership. For this reason, the third release of CFTI was published in English, backed by a set of articles discussing in detail its basic principles^10^.

Twenty-four years after the first version and twelve years after publication of a web-GIS version extended to the whole Mediterranean area^11^, here we present CFTI5Med, a largely revised and updated version of the Catalogue. CFTI5Med features many new contents and a completely revamped, user-friendly web-GIS interface allowing the Catalogue data to be analysed along with vitually any relevant georeferenced geological, geophysical and administrative data^12^.

## Methods

Our Catalogue hence draws from an extremely valuable and unique documentary and historical heritage^13^: one of the most important in the world, both in terms of quantity, quality and geographic distribution of the available information, and in terms of the relevant chronological interval, spanning more than two millennia (from the 8^th^ century B.C. to the 15^th^ century for Mediterranean area^14–16^; from the 5^th^ century B.C. to the 20^th^ century for Italy^11,12^).

The Catalogue brings together the results of research conducted by over a hundred historical researchers and some geophysicists since 1983. Their work was coordinated within specific research projects aimed at exploring the known and unknown seismicity of the areas selected by the booming nuclear industry. The research was systematic, centrally coordinated and conducted with a stable methodology, with stringent standards and with a specialised historical approach, specific to every investigated area and historical period (on basic data typology and taxonomy^17^). This guarantees considerable data quality and homogeneity, and maximises the transparency of any interpretative decision-making concerning the retrieved parameters. This approach has been further developed and elucidated in a Guide to Historical Seismology published in 2009 by Guidoboni & Ebel^18^.

As a result of all these circumstances, the Catalogue comprises the only Italian thematic historical compilation backed by over 25 years of research (1983–2007) conducted with uniform methods and goals. It is also a unique example worldwide of a catalogue whose main information basis consists of historical sources, carefully interpreted and made readily accessible to everyone.

**Fig. 1:**
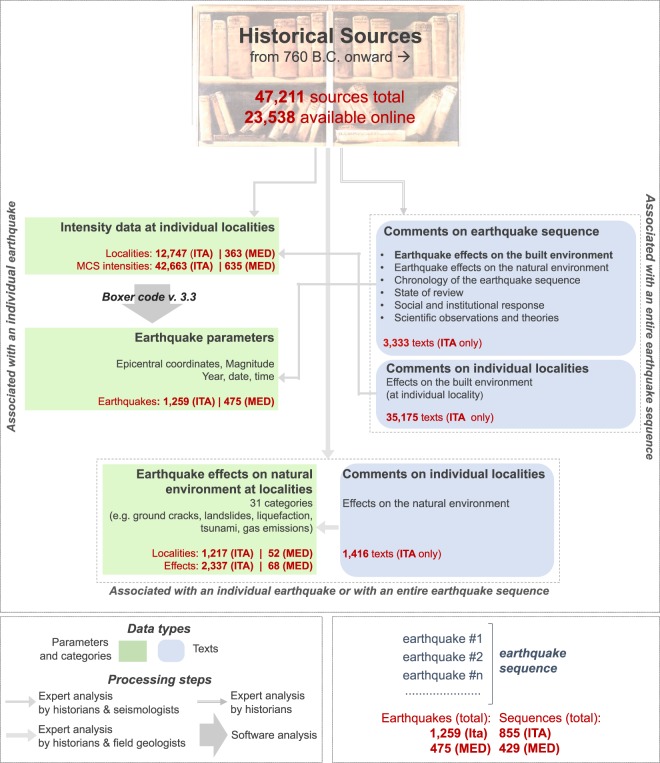
Workflow of the CFTI5Med (2018) release of the *Catalogo dei Forti Terremoti in Italia* (Catalogue of Strong Earthquakes in Italy). The scheme integrates two independent ways of describing the database contents: the larger titles in boldface describe its very structure, whereas the arrows describe how the available information was elaborated (see text for further details). Small red labels summarise the amount of information contained in CFTI5Med. Key: ITA, events located in Italy, or in the Mediterranean area but causing effects also in Italy; MED, events causing effects only outside Italy.

For all of these reasons, modern Historical Seismology is an inherently multidisciplinary field (Fig. 1**)**. In order to cope with this diversity the working group that developed the Catalogue includes historians, seismologists, earthquakes geologists, GIS and software experts.

### A new generation catalogue: the rationale

Although the Italian earthquake record is indeed an outstanding source of information, it does carry the inherent danger - and the paradox - of generating a misleading picture of the seismogenic potential of any area, often leading to an underestimation of the earthquake potential, but occasionally also to an overestimation; in both cases the misrepresentation of the earthquake potential may carry significant consequences, especially in areas undergoing fast economic development. Why is it so?

Until recently, the results of historical research have been summed up in traditional *parametric catalogues*, a form of presentation that indeed provides the basic data required for the elaboration of conventional seismic hazard models, but also inevitably spoils much of the available information. In parametric catalogues, the severity of earthquakes of the pre-instrumental era was assessed by reference to the maximum intensity only. Basic principles of earthquake source physics show that two crustal earthquakes of magnitude 6.0 and 7.0 may cause the same peak acceleration and the same maximum damage effects; but the area of strongest ground shaking will be much smaller for the 6.0 magnitude quake with respect to the 7.0 event. In the absence of instrumental data, the relative importance of the two earthquakes can be understood only by drawing complete pictures of their dynamic effects and translating them into earthquakes intensities, as it is done systematically in the Catalogue; a type of representation that parametric earthquake catalogues, focusing only on epicentral intensity, failed to supangaly.

As discussed in detail in the special issue of *Annali di Geofisica* edited by Boschi *et al*. in 2000^10^, the Catalogue proposed an entirely new approach to dealing with research on past earthquakes and to systematising the outcomes of the investigations. In addition to the standard source parameters, for each earthquake the Catalogue supplies a set of specifically prepared summaries, details on the effects suffered by each locality involved, and a list of references. It is hence an *analytical catalogue*, supplying all the information available for any given earthquake in a pre-defined and easily accessible format, including its effects on the social, built and natural environment; a wide spectrum of data and observations, some of which are not immediately relevant to seismic hazard applications, but may be of interest for broader seismic risk analyses and for many other applications. For every investigated earthquake sequence the Catalogue supplies also the relevant bibliography in an organised form, allowing the reader to navigate upstream from the parameters of a specific earthquake to the original sources that were used to investigate that event.

In summary, the information supplied by the Catalogue is both parametric and descriptive. The parametric information is similar to that supplied by conventional catalogues: conversely, the descriptive information on the earthquake effects and on their historical and administrative framework is unique to the Catalogue and, as far as we know, it is not supplied by any other earthquake catalogue worldwide. In fact, parametric data are used almost as an index to the non-parametric information. In this context, for instance, the assessment of the earthquake intensity at a given site takes on the role of a suggestion rather than of a rigid interpretation. The suggestion is generally qualified and motivated, but the user is free to develop his/her own evaluation by navigating back to the original information upon which the intensity assessment was based.

### General structure of the Catalogue

Figure 1 shows a schematic overview of the Catalogue and of the different processing steps performed to build it, starting from the retrieval and analysis of the historical sources. For each investigated earthquake sequence CFTI5Med supplies also a bibliography of all relevant historical sources.

Instrumental catalogues generally supply one record per earthquake. In contrast, the earthquake effects described by historical sources often refer to the combined effect of multiple shocks belonging to a sequence. This is generally unavoidable, as it is always difficult to separate the effects of subsequent shocks; either because they occurred very close in space and time, or simply because of the limited resolution of historical sources.

For all of these reasons, the Catalogue presents the data organised “by earthquake sequence”, after aggregating shocks that appear reasonably clustered in both time and space. Occasionally this aggregation has been quite difficult, in particular when two or more spatially and chronologically close sequences cannot be separated with confidence. The aggregation of multiple shocks into a single sequence has been crucial during the acquisition, filing and digitization of the retrieved historical information.

To facilitate access to the historical information and prevent any loss of data that are not directly used to describe the earthquake effects, but that may be useful to reconstruct the seismic scenario from different viewpoints, we chose to describe each sequence through a set of synthetic historical-critical comments. Conversely, earthquake parameters have been compiled for each individual shock, and so are the intensities reported at each locality. This dual “per individual earthquake” and “per sequence” approach is summarised in the left- and right-hand side of the scheme shown in Fig. 1, respectively. An exception is represented by the earthquake-induced effects on the natural environment; they are associated either with an individual earthquake or with an entire sequence, and therefore they are shown centred in the bottom section of the scheme.

**Fig. 2:**
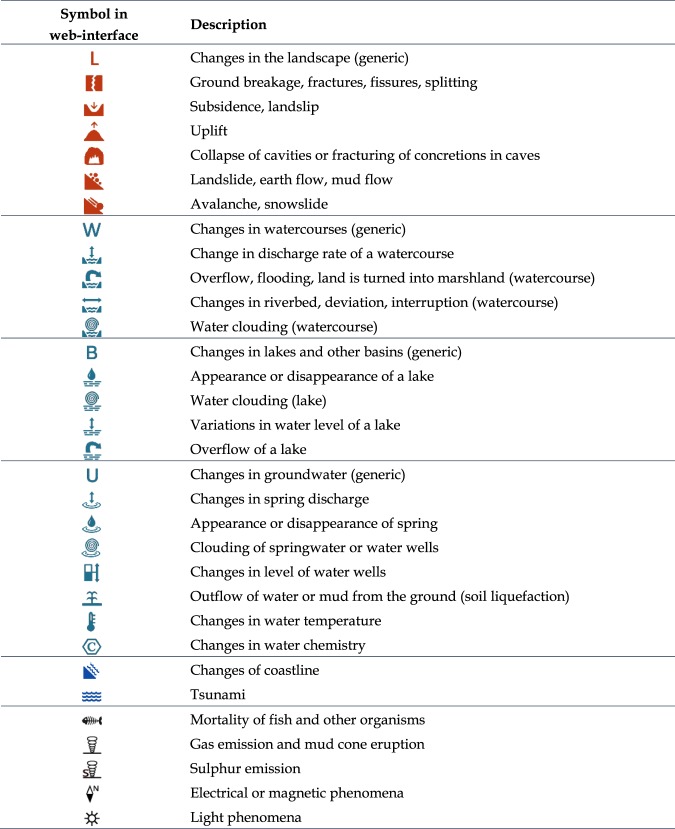
Classification of earthquake-induced effects on the natural environment adopted in CFTI5Med.

Hence, for each investigated earthquake sequence we provide three different types of parametric information (Fig. 1):

1. the full parameters of each shock of the sequence that can be analysed individually (see complete list below);
2. the intensity (according to the Mercalli-Cancani-Sieberg scale, or MCS scale) assigned to individual localities where each shock caused damage, or was simply *felt*, or was reported *not felt* (see complete list below);
3. a list of effects on the natural environment associated with a single shock (whenever possible) or with the entire sequence, subdivided into 32 different categories (Fig. 2) and assigned to individual localities.

Following is a list of the parameters supplied for any earthquake listed in the CFTI5Med:

- **Date:** earthquake origin date (year-month-day)
- **Time:** earthquake origin time expressed as Greenwich Mean Time (hour:minutes:seconds)
- **I**_**0**_**:** epicentral intensity (MCS)
- **Imax:** maximum intensity (MCS)
- **NMO:** Number of Macroseismic Observations for the given earthquake
- **Me:** equivalent magnitude based on macroseismic observations
- **Epicentral area**
- **Notes:**

F: false

D: doubtful

S: epicentral parameters based on a single intensity datapoint

E: epicentral location from foreign catalogue

N: no macroseismic observations available

- **RL:** review level of the study (low, medium, high)
  Following is a list of the parameters supplied for any locality affected by a specific earthquake listed in the CFTI5Med.
- **Is:** MCS intensity at site, for the given earthquake (with parameters listed below). In addition to the degrees of the MCS scale, in some instances other letters are used, based on the following list:
  - S(V) strongly felt, but lacking evidence to support or deny the occurrence of damage
  - F(IV-V) felt
  - NF not felt
  - G generic indication of damage at a specific site
  - N no evidence found in contemporary sources
  - NC unrated: please refer to the analytical comments for further info
  - *Effects on a single building*:
  - A(IX) collapse or extensive damage to the load bearing walls
  - B(VIII) collapse of the top portion of the building (lantern, dome, gable, etc.)
  - C(VII) partial collapse of the roof, vaults, apsidal vault, etc.
  - D(VI) falling eaves, cracking of the external walls
  - E(VI-VII) report of generic damage to the building
- **Nat:** earthquake-induced effects on the natural environment associated with a single earthquake or with the entire earthquake sequence
- **Date:** earthquake origin date (year month day)
- **Time:** earthquake origin time expressed as Greenwich Mean Time (hour:minutes:seconds)
- **I**_**0**_**:** epicentral intensity (MCS)
- **Imax:** maximum intensity (MCS)
- **NMO:** Number of Macroseismic Observations for the earthquake
- **Me:** equivalent magnitude based on macroseismic observations
- **Epicentral area**

In addition to the above parameters, we supply the following synthetic summaries (in plain text form):

- descriptions of the territorial impact and temporal evolution of the entire earthquake sequence;
- descriptions of the effects on the built and natural environment for each individual locality.

The MCS macroseismic scale was adopted as the most appropriate for evaluating the effects of damaging earthquakes of the past. The reader may refer to Ferrari and Guidoboni^19^, for a discussion of the reasons of this choice and for a thorough description of macroseismic scales.

**Fig. 3:**
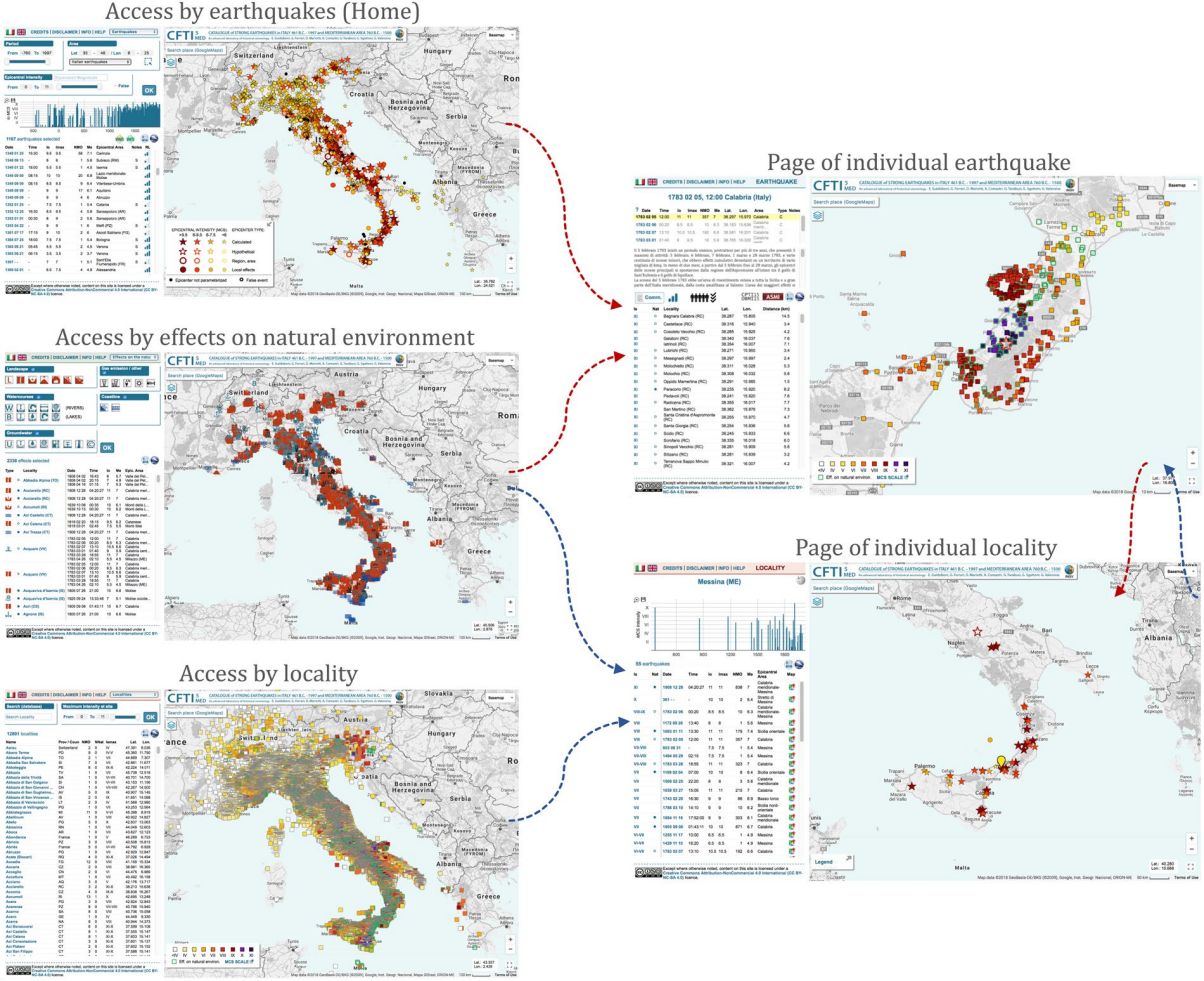
Synoptic view of the three main access modes to the data of the CFTI5Med, shown on the left. The two frames on the right show specific pages dedicated to each earthquake and to each locality analysed in the Catalogue.

The contents of the Catalogue can be accessed from three independent entry points relating to earthquakes, localities and effects on the natural environment, respectively referred to as “Access by earthquake”, “Access by locality”, and “Access by effects on the natural environment”. They refer to the three types of parametric data shown in Fig. 1 (green boxes on left-hand side). In addition, detailed information on each earthquake and locality listed in the Catalogue is supplied in specific, dedicated pages (Fig. 3).

In the following we provide more detailed descriptions of the information made available by the Catalogue and of the methodology used to build it.

### Elaboration of earthquake data: parametric information

Earthquake parameters indeed comprise the core of the information supplied by any earthquake catalogue. They are used for a number of elaborations ranging from the construction of earthquake scenarios, to the calculation of earthquake productivity rates, to the derivation of empirical earthquake attenuation laws, to the calculation of the parameters of an “equivalent seismogenic source”. As we pointed out earlier, however, the parametric part of catalogue is just one of the possible products of the research.

#### Intensity data at localities

Determining the macroseismic intensity of historical earthquakes is made difficult by the need to classify descriptions that are inherently qualitative. Such descriptions generally lack standardisation as regards the information levels, that can be highly variable, and the semantic value of the statements. Furthermore, the inferred damage levels may change significantly in relation to the economic and building context of each individual earthquake.

All intensity scales were designed for classifying the effects of earthquakes contemporary to the observers. The intensity is assessed by direct comparison between the testimonies supplied by historical research and the descriptions assigned to each degree of the adopted scale. The quality of the assessment is hence strongly controlled by the resolving power of the testimony.

In the Catalogue all intensity values were assessed by identifying convergent patterns in contemporary sources alone. Sources are sometimes contradictory, however, forcing the analyst to use his/her own experience to assess the local intensity based on the significance of the effects reported by the historical sources.

It must be stressed that proper use of intensity scales involves a statistical assessment of the effects of the earthquake. In other words, a specific intensity level should be assigned only as an average of the observed behaviour of a number of independent buildings in a settlement (a village, a city, etc.). In some instances, however, and especially for the oldest earthquakes, the available historical information concerns mostly single monumental buildings (churches, cathedrals, castles, towers etc.). Under such circumstances the assignment of an intensity is replaced by the assignment of a code (e.g. A, B, C, D, E: see the list of the parameters supplied for any affected locality) to somehow preserve the information.

The reader is encouraged to refer to Ferrari and Guidoboni^19^ for a thorough description of the difficulties involved in the use of an earthquake intensity scale and of the criteria followed in the Catalogue for attributing an intensity to a given locality.

#### Deriving earthquake parameters

One of the most critical steps in the parameterisation of historical earthquakes is the assessment of their epicentral location. When a sufficient amount of individual intensity data-points is available, earthquake source parameters can be determined through an analytical technique, giving a statistical basis to highly subjective historical data^20^.

To parameterise all earthquakes listed in CFTI5Med we used the Boxer computer code v. 3.3^21^. Boxer uses intensity data to assess the location, moment-magnitude, physical dimensions and orientation of the source of large historical earthquakes. The macroseismic epicentre is calculated as lying in the middle of the spatial distribution of all sites that suffered the largest intensities. The magnitude, referred to as “equivalent magnitude” or M_e_ to clarify that is an inferred - non measured - estimate, is obtained based on the average epicentral distance (i.e., the radius) reached by each intensity level. As the magnitude is calibrated using 20^th^ century earthquakes for which both the intensity pattern and the moment-magnitude M_w_ are known with confidence, the M_e_ obtained using Boxer corresponds to the M_w_. It is well known that the epicentral intensity I_0_ decreases for an increasing hypocentral depth, and vice-versa. This implies that M_e_ may be substantially larger or smaller than the magnitude one could obtain from empirical laws using only I_0_, depending on hypocentral depth.

Boxer may be hence used also to describe the source of an historical earthquake in terms of an “equivalent seismogenic source”, i.e. as an oriented “box” whose length and width are obtained from the M_w_ through empirical relationships. The box is meant to represent either the actual surface projection of the seismogenic fault or, at least, the surface projection of the portion of the Earth crust where a given seismic source is likely to be located.

The very long chronological interval covered by the Catalogue - about 25 centuries for Italy - implies that earthquake locations may fall into different typologies, summarised in the following:

- calculated from a sufficient number of intensity data-points (at least three);
- taken from foreign catalogues;
- hypothesised, when the distribution of intensity observations does not allow a full calculation;
- set coincident with the location of a single site for which there exists an intensity assignment;
- set as lying in the middle of a region identified by the historical sources as the area that suffered the largest effects, without specifying the names of individual localities;
- unparameterised: this condition refers to an earthquake for which the available information is insufficient or too generic for deriving analytical parameters.

#### Earthquake effects on the natural environment

For the new CFTI5Med we specifically focused on earthquake-induced effects on the environment. This goal was accomplished by re-analysing and broadening the scattered information supplied in previous version. More specifically, all testimonies reporting earthquake-induced effects on the natural environment were systematically analysed, geo-referenced and filed. This complex work was backed by the expert judgement of the analysts, including historians and geologists^22^.

Earthquake-induced effects on the natural environment reported in CFTI5Med were classified into 32 categories belonging to 5 macro-groups (Fig. 2):

- changes in the landscape (including landslides, ground cracks, ground uplift or subsidence, etc.);
- changes in watercourses and lakes;
- changes in groundwater (including liquefaction effects);
- changes in the coastline (including tsunami effects and costal landslides);
- others, such as gas emissions and light phenomena.

The classification includes four additional generic categories (changes in the landscape, watercourses, groundwater, lakes) that are used when the available testimonies do not provide detailed enough descriptions of the phenomena.

Two of the most significant categories of earthquakes-induced effects are *landslides* and *surface faulting*, all of which fall into the first of the above groups. However, while landslides are often explicitly mentioned by the historical sources (with 527 testimonies out of a total of 2,337), surface faults are never mentioned as such, simply because the relationships between seismogenic faulting, surface faulting and ground shaking have been correctly recognised only in relatively recent times. As a result, any reports that may actually refer to genuine (i.e., tectonically-driven) surface faulting are included in the subcategory “Ground breakage, fractures, fissures, splitting” (553 testimonies). Unfortunately, deciding whether a single report of ground breakage corresponds to actual surface faulting or rather to other non-tectonic processes is extremely subjective. For this reason we decided to avoid the risk of over-interpretation of the reported evidence, leaving to the user the responsibility to explore its true nature.

The effects reported in CFTI5Med are located as points coincident with localities where the effects were observed. In some instances, where historical descriptions allowed us to do so, the effects were precisely located on the geographical spots where they were observed (mountains, rivers, etc.). While MCS intensities are in one-to-one relationship with localities for any given earthquake, natural effects are not, since more than one type of phenomena may have occurred at the same site. For this reason, for each locality we may provide a list of effect categories.

As anticipated above, the effects on the natural environment are associated with individual earthquakes (whenever this is made possible by the accuracy of the testimonies) or with an entire earthquake sequence.

### Elaboration of earthquake data: descriptive information

The descriptive information includes synthetic historical-critical comments concerning the whole earthquake sequence and summaries of the effects suffered by each locality (Fig. 3). All comments are in Italian, but can be translated on-the-fly using a built-in function based on Google Translate.

#### Historical-critical comments on the entire earthquake sequence

The descriptive information concerning the main features of the whole earthquake sequence was organised following seven key-areas, from which we may derive up to 16 sub-areas, depending on the quality of the information available for each earthquake (Table 1). The information supplied includes basic elements of the institutional response (financing, tax exemption, new building codes) and of the social response (emigration, depopulation, epidemics). For the largest reported earthquakes, such as those occurring in the central and southern Apennines and in Sicily, the comments also focus on changes in the settlement network such as abandonments, relocations and reconstructions. These data are often necessary for reconstructing the urban and territorial planning history in Italy and for highlighting the critical role of large earthquakes of the past in determining the current structure of the main urban centres and of the settlement network in the most earthquake-prone areas.

**Table 1:** Structure of the historical-critical comments supplied for every earthquake sequence listed in the CFTI5Med.

Area 1. Main features of the earthquake sequence	1.1. Chronological parameters	Complete chronology of all shocks for which memory is preserved.
Area 2. State of review	2.1. State of earthquake review	A description of the quality level of the revision made and of the main types of basic data used, either bibliographic or archival.
	2.2. Development of earthquake review	A section highlighting specific research problems, successful and unsuccessful archival selections made, and data typologies.
Area 3. Social and institutional response	3.1. Elements of local demography	Data on the population of the areas hit by the earthquake, to be used to evaluate the social impact of the event.
	3.2. Characteristics of the local building styles	Main elements of the building heritage: materials used, building techniques and state of preservation of the building stock.
	3.3. Administrative/historical affiliations and boundaries	Main elements of the administrative hierarchies, which may affect the effectiveness of the institutional response.
	3.4. Social and economic response	A summary of the immediate circumstances exerting the greatest effects on the economic context.
	3.5. Institutional and administrative response	Main institutional and administrative dynamics characterising the post-earthquake stage; projects for moving sites, tax exemptions.
	3.6. Reconstructions, relocations	Main elements characterising the stage of reconstruction; decisions to rebuild elsewhere, timing, quality of the interventions
Area 4. Scientific observations and theories	4.1. Scientific observations and theories	A summary of interpretations of the earthquake expressed by contemporary philosophers, naturalists and seismologists
	4.2. Technical/scientific observations and surveys	A summary of indications from expert reports on technical assessments of damage; scientific context of the earthquake.
Area 5. Earthquake effects on the built environment	5.1. Major earthquake effects	Summary of the main tremors, of the most damaged sites, of the extent of the area struck by the earthquake, and of the typology of the most significant damage
	5.2. Concurrent natural and man-induced destructive events	Indications of the concomitance with other natural events (e.g. cascading events) that may have altered the perception of the earthquake impact
	5.3 Effects on the built environment by individual locality	A summary of the effects for each identified locality, supplied along with the relevant bibliographical references.
Area 6. Earthquake effects on the natural environment	6.1 Characteristics of the effects on the natural environment	Brief description of the effects of the earthquake on the natural environment, if reported.
	6.2 Associated natural phenomena	Indications on any phenomena associated with the earthquake; unusual animal behaviour, light phenomena, electromagnetic variations etc.
	6.3 Effects on the natural environment by individual locality	Brief description of the effects of the earthquake on the natural environment at the given locality.
Area 7. Bibliography		For every investigated sequence, CFTI5Med provides the relevant bibliography in an organised form. Each available witness has been filed individually to allow it to be easily retrieved. See the following section for further details on how the database deals with the supporting bibliography.

#### Historical-critical comments on individual localities

The descriptive information of the effects suffered by each locality following a specific earthquake is organised into two types of historical-critical comments:

- effects on the built environment: summary of the effects suffered by the given locality, including the number of victims, the number of collapsed/damaged buildings, the nature of damage, the institutional response, etc. They describe the effects of the entire earthquake sequence;
- effects on the natural environment: brief description of all earthquake-induced effects on the natural environment reported for the given locality. They can be associated with a single shock or with the entire earthquake sequence. While the parametric data on natural effects have a many-to-one relationship with each locality, the comment for each locality describes all of them and has therefore a one-to-one relationship with the locality.

**Fig. 4:**
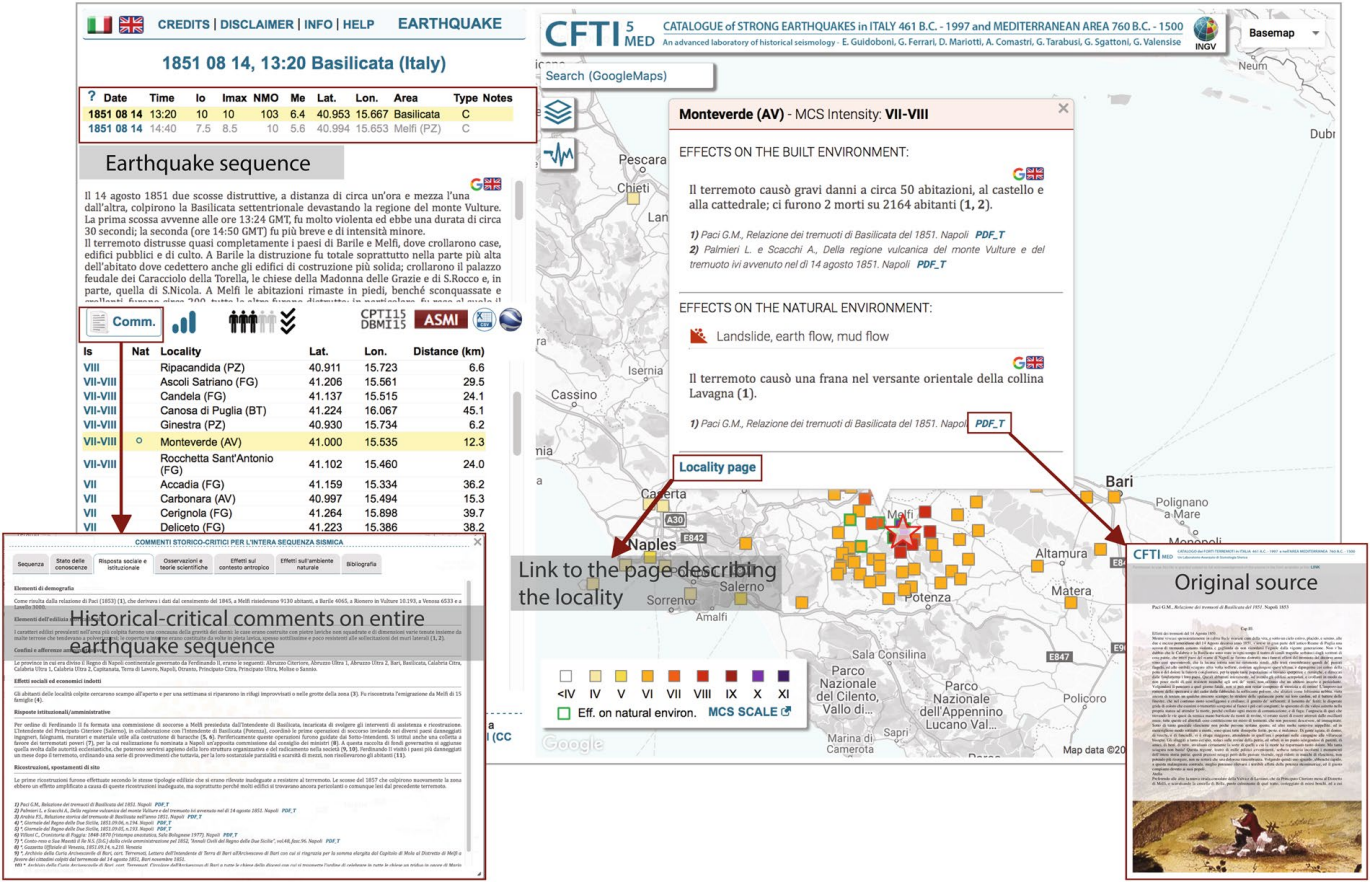
Summary of the capabilities of CFTI5Med and its web interface. The upper left panel shows the interrogation of a specific event of interest of the user, the 1851 earthquake sequence in southern Italy. The large infowindow on the right-hand side of the figure shows the geographic distribution of available intensity datapoints. The panel on the bottom-left shows the synthetic comments available for the same sequence. Selecting the locality “Monteverde” on the main panel to the left opens a smaller infowindow that describes the damage suffered by that locality as a result of the 1851 earthquake sequence plus any known effects on the environment. The same panel allows the user to open the PDF version of a paper referenced as describing such effects. The “Locality page” button in the lower left of the locality infowindow opens a new window detailing all the earthquakes reported in CFTI5Med that have been felt in Monteverde through history.

All comments can be accessed in the web-interface through informative windows that are displayed when selecting a specific locality listed in the “Access by earthquake” page, or a specific earthquake listed in the “Access by locality” page (Fig. 3). References to the relevant bibliography used to compile the summaries are listed below each text, followed by a link to the corresponding PDF file, when available (Fig. 4).

#### Assessing the number of earthquake victims

The number of victims caused by an earthquake is an extremely useful piece of information, particularly for civil protection planning, but is also one of the most unstable elements that can be derived from historical testimonies. Its accuracy is largely a function of the historical period considered and of the reliability of testimonies. For the oldest earthquakes these testimonies are often fraught with symbolic meanings and generally report unreliable estimates.

In the absence of official estimates, testimonies are often discordant and return very large intervals for the actual number of victims. For all of these reasons we decided to subdivide the number of victims into five large ranges (<10, 11–100, 101–1000, 1001–10.000, >10.000), attributing to each estimate a reliability class ranging from 1, lowest, to 3, highest.

When the number of victims is described non-numerically (i.e., using one or more adjectives), we interpreted the available testimonies in view of the presumed number of inhabitants of the cities or villages involved. Notice that this number is not very accurate even for Italy, where official census data became available only after the unification of the country in 1861 (although there are important exceptions also for much earlier times).

At any rate, the number of victims is hardly a robust indication of the earthquake territorial impact, as it depends on a combination of circumstance which include the date and time of the largest shock(s), the prevalent occupation of the population, and the occurrence of any foreshock(s) that may have forced everyone to leave all buildings and seek refuge in open spaces.

### Organization and classification of bibliographical sources

The bibliography comprises the foundation of a compilation and database dealing with historical natural phenomena. It includes both published and unpublished sources: when listed by author the unpublished sources were ordered by the name of the site where they are kept: one of 465 archives and 320 libraries in Italy, plus about a dozen libraries abroad.

All texts are classified based on the strength assigned to the testimony(es) they contain with respect to the earthquake sequence being considered (Table 2): notice that this classification refers to individual pieces of information relative to each sequence, not to the whole of the text. A classification of the strength of the individual testimonies was developed by ranking them according to their characteristics, and ultimately according to their reliability. Each one was assigned a specific code that allows the reader to assess more easily their potential relevance.

**Table 2:** Classification of bibliographical sources based on the strength assigned to the testimony(es) they contain with respect to the earthquake sequence being considered.

Direct source	The text was written by a contemporary witness, both trustworthy and geographically close to the event described, whose testimony is hence deemed reliable and authoritative.
Source chronologically close to the event	The author is chronologically close to the event (lifetime), but is geographically distant from the site to which the testimony refers. It includes all cases where the witness was obtained indirectly from another text or by oral tradition, and it is impossible to trace back the path followed by the information.
Indirect source	It may include: - texts of local historiography which are deemed especially authoritative and close to the event; - especially authoritative narrative sources; - journalistic texts of the time.
Apocryphal source	It is said of a text that was deemed unreliable. Texts of this category are included in the bibliography only because they were used in previous studies and as such deserve to be re-considered.
Repertoire (pre-modern catalogues)	A list of earthquakes prepared according to the viewpoints of literary and naturalistic erudition. It also includes texts on the “freak events” that are typical of the beginning of the modern age, and in general all catalogues compiled before the nineteenth century.
Repertoire-type source	This expression refers to direct sources of information contained in a Repertoire (see above), which in itself can not have the value of a true source since it is usually a collection of late and indirect information.
Catalogue	List of earthquakes compiled from the nineteenth century onwards for scientific or naturalistic purposes. It may be based on selections of sources or works quoted, or may not report any indication of the sources used. It may be descriptive or parametric.
Catalogue-type source	It is a direct testimony contained in an earthquake catalogue, chronologically contemporary to the author or drawn up from direct observations by the author in person or transcribed in full from original texts.
Bulletin	A publication produced through observations or recordings by specialist centers; a table of measurements; a complete or even partial series of measurements taken within different contexts (deeds of academies, articles, etc.). Observations of earthquake effects reported in observatory registries have been considered direct or indirect sources, depending on each specific case.
Generic information	This definition is used for information taken from secondary historiographic sources (e.g. local social histories), in coeval memoirs or in antiquarian historiography, when the event described is very distant in time or when the information is so generic that is reasonable to assume that the data are not supported by sources known to the author. This code indicates that this information requires further verification, or that it may be interpreted only as a clue for further investigations.
Historiographic study	This code includes studies by contemporary historians (but not necessarily conducted using the criteria of recent critical historiography), specific studies on past earthquakes or studies on relevant aspects of the economic, demographic and social history that were used to improve the understanding of the earthquake effects.
Scientific bibliography	This code is assigned to coeval studies conducted within a specific theoretic interpretative and cognitive framework, starting with the beginning of the nineteenth century, or to recent studies on past earthquakes.
Negative source	Source that does not contain information on the earthquake sequence.

For further information on this topic the reader is encouraged to refer to the Info section of the Catalogue.

### Current limitations and perspectives

As with any compilation of historical information, and despite our best efforts, the data supplied in the Catalogue are subject to various limitations. The worst possible limitation is the lack – or extreme paucity - of the available information, either because no source has supplied any testimony on a specific earthquake, or because the available testimonies have been lost. If only one source is available, the Catalogue will report it as evidence of the occurrence of an earthquake, but it will be nearly impossible to locate it and assess its magnitude, suggesting much caution in making any inferences that may affect public safety.

There are at least two large areas of the Catalogue that could be improved in due course. The first area concerns a number of large medieval earthquakes generated by the Apennines seismogenic zones. There is room for a substantial improvement of their characterisation, based on available written sources but also on archaeoseismological evidence and on the new data arising from the restoration of damaged monumental buildings.

The second area concerns the reconstruction of damage scenarios in art heritage cities, an important aspect of tourism-based economy. Further investigations of the damage suffered by monumental buildings would provide crucial information on the seismic vulnerability - and hence on seismic risk – for invaluable cities such as Venice, Florence, Rome and Naples, among many others.

More specifically, in at least three instances the Catalogue itself supplies an indication of what could be achieved with further analyses:

1. each earthquake is rated in terms of the quality of the investigations conducted to describe and parameterise it. The rating, expressed in terms of *Revision Level* (see RL and the adjacent field Notes in the “Access by Earthququake” page) and ranging from “low” to “medium” to “high”, indicates at first glance which earthquakes could be investigated in better detail;
2. the Catalogue lists several tens of earthquakes that in its previous version were labelled “unknown”, to indicate that they were not reported in previous catalogues. In CFTI5Med they have been fully integrated in the earthquake list and rated “low level”. For most of them, however, there is ample room for further investigations, so that they could be taken to “medium level” or “high level”;
3. so far the chronology of earthquake sequences has not been investigated in detail, and in most cases only the mainshock has been looked at in detail. This trend has already been reversed by previous versions of the Catalogue, and there is room for obtaining intensity data for individual large aftershocks, thus improving the resolution of the territorial impact of the earthquake sequence.

## Data Records

CFTI5Med stores information on 1,259 earthquakes (98 of which are currently considered false) that occurred in Italy from 461 B.C. to 1997, and 475 earthquakes that occurred in the extended Mediterranean area (outside Italy) from 760 B.C. to 1500. A summary of all the information contained in CFTI5Med is given in Fig. 1. In addition to being available on an INGV website (10.6092/ingv.it-cfti5) the full dataset is published in the PANGAEA® Data Publisher^23^ with the following structure:

- a table supplying the source parameters for all individual earthquakes listed in the Catalogue;
- a table supplying MCS intensities at localities for all individual earthquakes listed in the Catalogue;
- a table supplying all known earthquake-induced effects on the natural environment for all individual earthquakes listed in the Catalogue;
- a table supplying names and geographical coordinates for all localities listed in the Catalogue;
- a set of XML files, one for each earthquake sequence, containing descriptive information (in plain text) and bibliography for the entire earthquake sequence and for each locality.

The connections between the different tables are based on univocal codes for each earthquake sequence, for each individual earthquake and for each locality listed in the Catalogue.

In addition to downloading data from the PANGAEA® repository^23^, the user may download data directly from the CFTI5Med web-site^12^. From each type of page of the web-interface (Fig. 3) the user may access the parametric information, listed in tables that can be downloaded as CSV (Comma Separated Values) or KML (Keyhole Markup Language) files. In particular, the user is allowed to download the following tables:

- earthquakes listed in the Catalogue, one record per earthquake. The number of records depends on the query selected by the user;
- localities listed in the Catalogue, one record per locality. The number of records depends on the query selected by the user;
- effects on the natural environment listed in the Catalogue;
- effects of each individual earthquake listed in the Catalogue;
- earthquake history of any individual locality listed in the Catalogue.

All map locations are expressed by their geographical coordinates in degrees (according to (WGS84), rounded to three decimal places.

## Technical Validation

Of all possible earth science datasets, an earthquake catalogue is indeed one of the most difficult to validate. This statement applies fully to a compilation of historical earthquakes such as that presented here, but also to a catalogue of recent seismicity recorded by state-of-the art seismological networks. Earthquakes occur in the Earth crust following a sort of “random regularity”: they concentrate in areas that we refer to as “seismogenic”, but occasionally visit areas where the geodynamic activity of our planet is comparatively slower, making seismic activity appear more sporadic. On the one hand, past earthquakes tell us where we may expect future earthquakes to strike; on the other hand, contemporary earthquakes, which by definition are better located than those of the pre-instrumental era, somehow validate past seismicity. There are important exceptions, however, because large earthquakes are also rare, and may occur in areas where background seismicity is limited or apparently absent, at least during the short time interval - a few decades - resolved by instrumentally recorded data.

There is clearly no experiment that can be planned to formally validate an earthquake database, but there are ways to evaluate empirically the accuracy of the data presented. Most large European earthquakes have been investigated by more than one group or scientists, each of which generally elaborated a map of the intensity pattern and derived an epicentral location and a magnitude. The European initiative AHEAD^24^ (European Archive of Historical EArthquake Data: https://www.emidius.eu/AHEAD/index.php) collected all solutions made available in the literature for a number of significant European earthquakes. For instance, the great 25 January 1348 earthquake is a catastrophic event that destroyed a large region at the boundary between present-day Austria, Italy and Slovenia. From the relevant web page on AHEAD (https://www.emidius.eu/AHEAD/event/13480125_1530_000) we learn that the earthquake currently appears in seven earthquake catalogues and was investigated by an Italian group^11^, by an Austrian group^25^, and by seismologists of the French BRGM (http://www.sisfrance.net). It follows that the variability of the epicentral locations, indeed quite large (about 50 km in latitude and 30 in longitude), and the equally large range of the assigned magnitudes (6.4–7.1) may be taken as an indication of the uncertainty associated with the parameters of this earthquake. Incidentally, the study presented in CFTI4Med was selected as the most reliable by the compilers of AHEAD, who in this case acted as an independent authority.

Over the past 20 years the Catalogue has been the main source of data for the CPTI catalogue^9,26^, that is the reference dataset for any seismic hazard elaboration in Italy. Such an implicit endorsement is important, also considering that historical seismicity data are constantly challenged by observations of different origin, from geological to seismographic to GPS.

Finally, it should be pointed out that unlike standard earthquake catalogues, our Catalogue allows the basic data - essentially earthquake intensities - to be accessed and re-evaluated by the user. This peculiar characteristic of the Catalogue necessarily reduces the emphasis on the validation of earthquake data.

## Usage Notes

The contents of the Catalogue are fully accessible to all users through an efficient and user-friendly web- and web-GIS interface, whose general structure is shown in Fig. 3). More specifically:

- the page “Access by earthquakes” (website home-page) allows the user to display the list of all earthquakes contained in the CFTI5Med database and make queries concerning a specific time interval, epicentral intensity/equivalent magnitude interval, or area;
- the page “Access by locality” allows the user to display all localities, search among them and make queries based on a maximum intensity interval (the maximum intensity is the largest value reported for a given locality in the Catalogue);
- the page “Access by effects on the natural environment” allows the user to display all earthquake-induced effects on the natural environment and make queries based on the different categories of effects. Specific web-pages provide information on each individual earthquake and locality of the Catalogue (see “Page of individual earthquake” and “Page of individual locality” in Fig. 3).

All five types of web-pages have a similar structure (see Figs 3 and 4), consisting of an informative section on the left (a selection panel and/or tables and/or additional information) and a map on the right, showing markers of earthquakes/localities/effects on the natural environment/macroseismic observations, all listed in the table located in the left frame. This frame also hosts two icons that allow the selected data to be exported in plain text or KML format. The navigation through the web-interface is guided by contextual help messages that appear any time the mouse rolls over an icon, table header, query section, etc.

In all types of pages, all markers and some table elements are clickable. Upon clicking on them, an informative window (infowindow) appears on the map (connected to the corresponding marker) and the corresponding table line is highlighted in yellow. The infowindow contains information on the selected earthquake/locality/macroseismic observation/effects on the natural environment, plus direct links to the page describing an individual locality or an individual earthquake (Fig. 3).

The infowindows contain also historical-critical comments describing the effects of the earthquakes on the built and on the natural environment at the given locality, along with references to relevant bibliography. Instead, the historical-critical comments describing the whole earthquake sequence can be accessed from the page of an individual earthquake by clicking on the “Comm.” button (Fig. 3). Here the user can also find a complete bibliographic list for the earthquake sequence. Each bibliographic source may be available in PDF form (Fig. 3), as explained in the following section.

All pages are available in Italian and English language. When the language is set to English, a button appears on the top-right corner of each historical-critical comment: by clicking the button the user may obtain a translation of the text by Google Translate.

For further details on how to navigate the CFTI5Med web-interface, the reader is encouraged to visit the “Help” section of the database website (10.6092/ingv.it-cfti5).

### Access to bibliographic sources in PDF

The Catalogue makes available as searchable PDF files 23,538 of the 47,211 testimonies used for compiling it. The PDFs may refer to papers, books, archival documents and letters that may be extremely difficult to find, or are kept in rather inaccessible libraries and repositories. The recovery and digitization of the bibliographic sources is an ongoing activity, and future versions of the database will feature a substantial number of new sources (in the order of a few thousands). Unfortunately, not all sources can be shown in the database: some are protected by copyright and can only be accessed by scientists, while others are stored in libraries and archives that do not allow the original materials to be reproduced.

In most cases the PDF files contain an exact transcription (PDF_T) of the excerpts that are deemed useful for further investigations, whereas in a limited number of cases, and only for texts that were originally in printed form, they contain scanned, searchable images of the original sources (PDF_R). For texts written in languages other than Italian, such as ancient and medieval Mediterranean languages (Latin, Greek, Syriac, Arabic, Hebrew), or in modern European languages, the transcripts contain a literal translation into Italian.

In some instances the Catalogue supplies both the PDF_T and the PDF_R files for the same testimony: while the transcript contains a faithful excerpt that may be useful for defining the full scenario of the macroseismic effects, the scanned version of the original source allows all historical-critical, geological and seismological contributions to be consulted in full (including for example a geological or seismological introduction, drawings, photos, maps, scientific interpretations and theories). Each PDF file contains the text and its exact bibliographical reference: information on how credit should be given is supplied in the PDF header.

### Interactive access to external datasets

Taking advantage of the most advanced public-domain web-GIS tools, the CFTI5Med web-interface allows its own georeferenced data to be plotted along with data obtained from a number of external resources, including administrative, topographic, tectonic, geological, geotechnical and seismological datasets (see list below). The external data are shown as map overlays chosen through selection panels that the user can access by hitting two buttons located on the top-left corner of the map.

This new possibility allows the user to consult the Catalogue data in the context of other relevant pieces of information, thus improving the ensuing interpretations. Examples of this opportunity include:

- plotting intensity data for a specific earthquake along with topographic, geological or landslide data, to verify whether specific geomorphological and geological circumstances may justify apparently anomalous intensities;
- plotting historical seismicity data along with instrumental data from an ongoing earthquake sequence, to verify the spatial relationships between the current earthquake locations and the areas affected by large earthquakes of the past;
- plotting the intensity pattern of a specific historical earthquake along with the distribution of mapped faults or seismogenic sources, to verify if the given earthquake may be interpreted as a result of the activation of a known fault/seismogenic source;
- plotting intensity data for a specific earthquake along with administrative boundaries, settlement distributions and transportation networks. Such a comparison may serve different purposes; for example, verifying whether the extent of damage or the effectiveness of the post-earthquake recovery was affected by conditions pertaining to specific non-geological characteristics of the region hit by the earthquake.

There exist two buttons to add map overlays:

- the first (top) button opens a list of map overlays that can be displayed on the basemap (note that the availability of some layers is scale-dependent). This list currently includes:
  - Borders of Italian *Comuni* (from ISTAT, 2016);
  - Borders of Italian *Province* (from ISTAT, 2016);
  - Borders of Italian *Regioni* (from ISTAT, 2016);
  - IGM topographic map, 1:25,000 scale (from Portale Cartografico Nazionale – PCN);
  - IGM topographic map, 1:100,000 scale (from Portale Cartografico Nazionale – PCN);
  - DISS Individual Seismogenic Sources – ISS (from INGV-DISS website);
  - DISS Composite Seismogenic Sources – CSS (from rom INGV-DISS website);
  - DISS Subduction Zones -SZ (from rom INGV-DISS website);
  - Geological map, 1:100,000 scale (from ISPRA);
  - Landslide catalogue (from Portale Cartografico Nazionale – PCN), with a web-interface that can be displayed by clicking on the “Legend” button;
- the other button opens a window that allows users to add to the map the instrumental earthquake locations supplied in real-time by INGV’s surveillance service (http://cnt.rm.ingv.it: from 1985 onward), based on queries made through a selection panel. The epicentres are shown on the map after hitting the ‘OK’ button.

### Potential users

CFTI5Med may provide crucial data for a number of different applications. These data are easily accessible through the web-interface to potential users having different backgrounds and roles in the society. Potential users/applications include:

- civil protection officers/risk assessment and emergency planning and first response;
- local authorities/territorial planning;
- professionals (engineers, geologists, architects)/consultancy on buildings, on infrastructures and on cultural heritage;
- professionals (various)/urbanistic evolution of towns and cities located in earthquake-prone areas;
- researchers (e.g. geoscientists, historians)/development of the reference national seismic hazard model (e.g. see http://zonesismiche.mi.ingv.it), identification of suitable scenario earthquakes, investigation of specific seismogenic sources, studies on the economic, social and demographic trends of a region struck by a large earthquake^27,28^;
- general population/raising risk awareness.

Users may hence take advantage of the Catalogue “in bulk”, i.e. by downloading large portions of it and analysing them with automated procedures, for example to derive earthquake magnitudes; or they may access individual portions of the information supplied, for example to investigate in detail the effects of a specific large earthquake in a locality of interest.

We wish to remark that the method developed to construct the Catalogue, its current architecture and its software could rather easily be adopted by countries other than Italy. Every earthquake-prone country has its own ways of dealing with the historical earthquake record, depending on the nature and quality of the available historical sources and on the level of scientific interaction among historians, seismologists and earthquake geologists. Developing a Catalogue similar to CFTI5Med would be straightforward for several countries of the central and eastern Mediterranean area, that could benefit from the knowledge that already exists on earthquakes that occurred in the Antiquity and up to the 15^th^ century. Nevertheless, we envision a possible extension of the Catalogue to countries of ancient civilization such as China and Japan, but also Indonesia and the Philippines, as well as to high-seismicity countries of the New World such as Chile, Argentina, Bolivia, Perù, Colombia and Venezuela.

## ISA-Tab metadata file

Download metadata file

## Data Availability

The custom code used to develop the CFTI5Med web-interface is entirely open and based on HTML language. As such the code can be reutilised by whoever is interested in replicating elsewhere our experience. The server-side procedures were developed in PHP open-source language. The client-side procedures were developed in JavaScript language, using specific features of jQuery (http://jquery.com/) and Google Maps API (https://developers.google.com/maps/) to provide a reliable and fast geographic interface. The Boxer computer code v. 3.3 used to derive earthquake source parameters is available online^21^.
